# Neural Coupling between Interhemispheric and Frontoparietal Functional Connectivity during Semantic Processing

**DOI:** 10.3390/brainsci13111601

**Published:** 2023-11-17

**Authors:** Takahiro Soshi

**Affiliations:** Department of English Language Studies, Faculty of Foreign Language Studies, Mejiro University, Shinjyuku, Tokyo 161-8539, Japan; t.soshi@mejiro.ac.jp

**Keywords:** neural coupling, functional connectivity, semantic processing, electroencephalogram

## Abstract

Interhemispheric and frontoparietal functional connectivity have been reported to increase during explicit information processing. However, it is unclear how and when interhemispheric and frontoparietal functional connectivity interact during explicit semantic processing. Here, we tested the neural coupling hypothesis that explicit semantic processing promotes neural activity in the nondominant right hemispheric areas, owing to synchronization with enhanced frontoparietal functional connectivity at later processing stages. We analyzed electroencephalogram data obtained using a semantic priming paradigm, which comprised visual priming and target words successively presented under direct or indirect attention to semantic association. Scalp potential analysis demonstrated that the explicit processing of congruent targets reduced negative event-related potentials, as previously reported. Current source density analysis showed that explicit semantic processing activated the right temporal area during later temporal intervals. Subsequent dynamic functional connectivity and neural coupling analyses revealed that explicit semantic processing increased the correlation between right temporal source activities and frontoparietal functional connectivity in later temporal intervals. These findings indicate that explicit semantic processing increases neural coupling between the interhemispheric and frontoparietal functional connectivity during later processing stages.

## 1. Introduction

Natural language processing is supported not only by the dominant left hemispheric areas but also by the corresponding right hemispheric areas [1,2,3,4,5,6,7,8,9,10]. Interhemispheric connections are domain-specifically observed mainly in the frontal and temporal language-associated areas [11,12] during high-load verbal processing, such as the comprehension of figurative language and complex contextual meaning [13,14,15,16,17,18]. Interhemispheric interaction is beneficial for high-load verbal processing, as it increases the neural resources for additional information processing [8,19,20,21]. High-load verbal processing also increases attentional burden and recruits frontoparietal connections for general cognitive function [22,23,24]. Although neural coupling between interhemispheric and frontoparietal functional connectivity (FC) may occur during high-load verbal processing, it is unclear how and when these FCs function interactively. The current study, therefore, examined the temporal interaction between interhemispheric and frontoparietal FCs for explicit semantic processing using a temporally resolved electroencephalogram (EEG).

The hypothesis for the current study was that high-load semantic processing increases neural coupling between cortical activity in the right hemisphere and frontoparietal FC. As argued for explicit information processing such as sensory perception [25,26] and verbal processing [19], cortical activation may initially occur in dominant modality-specific areas in a bottom-up manner and later spread into corresponding areas in the nondominant hemisphere [27] through enhanced frontoparietal FC under attentional control. Frontoparietal FC increases during explicit semantic processing, which may instantiate a general architecture for conscious information processing. Sustaining frontoparietal FC for maintaining information contributes to higher-order information processing in later information processing stages, such as judgement, memory encoding, and recall [26,28,29]. High-load semantic processing may increase frontoparietal FC, which is coupled with neural activity in the right hemisphere at later processing stages.

Dynamic FC is effective for investigating the neural coupling between FCs in different brain regions. FC represents neural synchronization between cortical or subcortical loci during cognitive processing, which can be estimated by the statistical correlation between temporally fluctuating signals [30,31,32]. Dynamic FC is based on the notion that FC dynamically changes in the time course of neural processing and is represented by, for example, a sliding-window correlation (SWC) [33,34,35,36,37]: The correlation weights are successively calculated for temporally overlapping sliding-time windows. This advantage of high temporal resolution of dynamic FC analysis is beneficial for elucidating the temporal characteristics of neural synchronization between interhemispheric and frontoparietal FCs.

The present study used secondary data of a semantic priming experiment to investigate the explicit and implicit processing of associated words stored in the long-term memory [38,39,40]. The experimental paradigm included two tasks with different attentional orientations. The explicit semantic processing task (EST) required participants to pay attention to and judge the semantic congruency between visually presented words. The implicit semantic processing task (IST) required participants to lexically judge whether a stimulus is a word. Although semantic priming effects, such as facilitated processing speed, appear without attention to semantic relations, as in the IST, the EST drives direct attention to semantic relations and requires more neural resources for semantic processing [41,42].

Cascade EEG analyses were conducted to examine surface brain potentials, cortical source activity, and dynamic FC between cortical loci. First, to confirm reduced negative event-related potential (ERP) effects for processing semantically congruent targets, a surface potential analysis was performed using noise reduction and signal separation analysis. Second, signal source estimation was conducted to examine cortical source activity (current source density: CSD) for semantic processing using standardized low-resolution brain electromagnetic tomography (sLORETA) [43]. Third, a dynamic FC analysis was applied to CSD data to examine frontoparietal FCs; frontoparietal FC was subsequently correlated with CSD in the language-related cortical areas in the right hemisphere to examine the neural coupling between interhemispheric and frontoparietal FCs for semantic processing. We predict that neural coupling between frontoparietal FC and right hemispheric cortical activity increases during the EST because it requires more neural resources for direct attention to semantic relatedness.

## 2. Materials and Methods

This study used the secondary data from the author’s previous research [44] to newly examine the neural coupling between right hemispheric activity and frontoparietal FC during explicit semantic processing. We did not newly obtain any data and informed consent and did not undergo a new ethics review. The sociodemographic and behavioral profiles of the 26 participants and ethical comments were already described in a previous study [44].

### 2.1. Experimental Tasks

This study focused on two semantic conditions (congruent and incongruent) from a previous study. A semantically congruent condition (SC) included congruent prime nouns preceding target verbs (“satou”/“tokasu” (sugar/melt)). As a baseline condition for comparison, we used a semantically incongruent condition (SI), in which incongruent prime nouns preceded target verbs (“gēmu”/“tokasu” (game/melt)). In the EST, 13 participants (20.7 ± 2.3 years) were requested to judge the semantic congruency between words directly, whereas, in the IST, another 13 participants (21.6 ± 3.7 years) judged whether the stimuli involved nonwords as soon as the targets appeared.

### 2.2. Experimental Material

Sets of 50 prime and target words each were included in the SC and SI. Prime nouns were the objects of transitive target verbs. The primes had three phonological units (mora), and their familiarity was confirmed using a 7-point scale (prime: 5.96 ± 0.32; target: 5.86 ± 0.26). The degree of semantic congruity between primes and targets was assessed using a 5-point scale (SC: 4.88 ± 0.18; SI: 1.53 ± 0.23). The second positions in the word sequences were occupied by unfamiliar pseudo-words (1.57 ± 0.02). The same prime–target sequences did not occur twice but appeared randomly in each of the four experimental sessions.

### 2.3. Experimental Procedure

**Figure 1:**
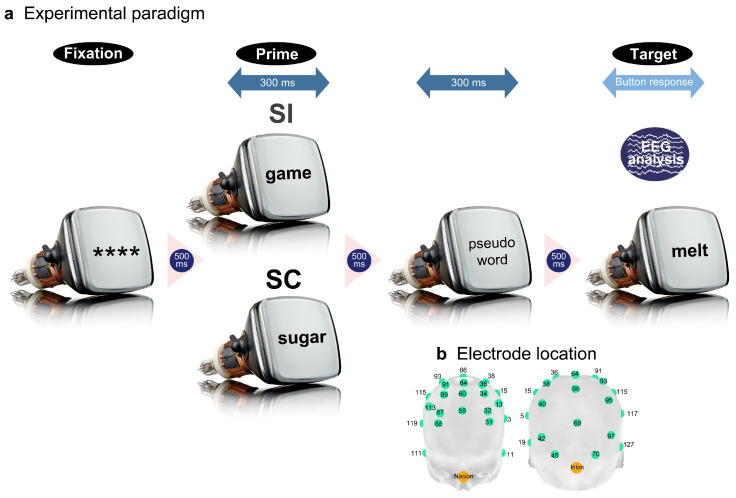
The experimental paradigm (**a**) and EEG recording locations (**b**). A priming paradigm was used to investigate explicit and implicit semantic processing. A prime word in Japanese was presented 500 ms after the fixation (****) appeared, for 300 ms. Subsequently, a pseudo-word appeared for 300 ms with an inter-stimulus-interval of 500 ms. Finally, a target word was presented with the same inter-stimulus interval and remained on the screen until the participants’ button response. The experimental stimuli comprised SC and SI prime–target pairs. EEG signals were recorded from the 34 scalp locations indicated by the numbered light-green nodes and were analyzed to evaluate neural responses to the target words.

The stimuli were presented to the participants via a 17-inch CRT monitor placed 0.7 m away inside an electrically shielded, soundproof room. A fixation symbol (****) first appeared in black in the center of the screen against a light-grey background (Figure 1a). The first and second stimuli were presented 500 ms after the preceding stimulus disappeared and were presented for 300 ms. The target verbs continued to appear until the participant’s response. The participants pushed the left or right button to choose the correct answers; the response mapping was counterbalanced across participants. The EST or IST were explained before the start of the experiment and were instructed to answer the questions as quickly and accurately as possible.

### 2.4. Data Recording

EEG signals were recorded by 34 Ag/AgCl electrodes on an electrode cap (Quik-Cap 128ch NSL; Compmedics Neuroscan, Charlotte, NC, USA), which were selected from 128 electrodes placed as evenly as possible (Electrode numbers: 3, 5, 11, 13, 15, 17, 31, 32, 34, 36, 38, 40, 42, 59, 60, 62, 64, 66, 68, 70, 71, 86, 87, 89, 91, 93, 95, 97, 111, 113, 115, 117, 119, 127; Figure 1b). Electrooculography (EOG) was performed using three electrodes placed around the eyes. All electrodes were referenced online to the left mastoid and offline to the linked mastoid. The ground electrode was placed on the anterior prefrontal surface. The data were recorded with a sampling frequency of 250 Hz and were amplified with a band-pass frequency from 0 to 70 Hz, and the impedance level was maintained under 5 kΩ.

### 2.5. Surface Potential Analysis

The stored EEG data were segmented into 50 epochs from 200 ms before to 800 ms after the presentation of the target words. Subsequently, EOG artifacts were removed from each epoch using the regression method (raw EEG*_ij_* = β*_ij_* × EOG*_i_* + e*_ij_*; estEEG*_ij_* = raw EEG*_ij_* − β*_ij_* × EOG*_i_* + e*_ij_*; *i* = numbers of EEG epochs; *j* = numbers of channels; estEEG: estimated EEG; e: noise) [45]. The EOG data were derived by subtracting the amplitudes at the lower-left electrode from those at the upper-left electrode. Subsequently, the epochs were filtered by forward and inverse fast Fourier transforms with bandpass frequencies ranging from 1 to 30 Hz with a frequency resolution of 1 Hz. The filtered epochs were baseline corrected using the mean amplitudes of the baseline interval (−200 to 0 ms). Epochs contaminated with residual artifacts, such as baseline drift, were discarded from following analyses based on the absolute amplitude threshold > |±50| μV. We used this strict rejection criterion set to a lower limit of EOG amplitude [46] because EOG reduction was applied to raw EEG in advance. One participant, who had a rejection rate > 30%, was not included in the subsequent analyses for both the EST and IST. The mean rejection rates of the participants were approximately 1% and 17% for the EST and IST, respectively. Individually averaged ERPs from 12 participants were obtained for each task.

We applied principal component analysis (PCA) [47] to each participant’s averaged ERP time-sequence data (−200 to 800 ms; 250 points). PCA is a blind source separation method used to obtain latent original signals (here, cortical source activities) and generally comprises the following procedures: (i) subtraction of the mean amplitude from the ERP data, i.e., centering; (ii) calculation of covariance matrices of the centered data; and (iii) eigenvalue decomposition of a covariation matrix. Using these processes, we obtained 34 time-sequence components, which were equal to the number of scalp electrodes and represented by eigenvalues or weighted coefficients (34 ERP components × 34 coefficients). The first and second dominant components were retained to reconstruct the ERP waveforms.

To elucidate the spatiotemporal patterns of ERPs, 34 scalp electrodes were hierarchically clustered based on the amplitude effects of semantic congruency (SC minus SI) in each task. The difference in amplitude between the paired electrodes (200 temporal points × 34 electrodes) was converted into a Euclidean distance. Most adjacent electrodes or electrode clusters were paired using the Ward or centroid method until 34 electrodes converged into a single cluster. As four superordinate clusters were similarly observed for the EST and IST, the ERP data were averaged across electrodes within each cluster separately for the SC and SI in each task.

The average amplitudes were compared between the SC and SI using a paired permutation *t*-test. The ERP waveforms for the SC and SI were successively averaged over 20 ms (five data points) throughout the intervals using a moving average method with a 4-point overlap. For each time window, SC and SI were compared to obtain dummy *t*-values (*n* = 245) for both the EST and IST. Dense comparisons (245 time windows × 4 clusters = 980 comparisons for each task) may induce type I errors without correction or type II errors with family-wise error correction. Therefore, we used a permutation test [48,49,50], in which the tested probability distributions of *t*-values were empirically obtained by multiple comparisons of samples randomized across conditions and participants. The total data (245 time windows × 4 clusters × 12 persons × 2 tasks = 23,520 samples) were initially randomized, and 12 samples were recollected for the SC and SI and compared to obtain dummy *t*-values. The resampling procedure was repeated 30,000 times to produce a permutation distribution. Actual *t*-values (*n* = 980) were tested on the criterion of significance of the α level of *p* < 0.05, corrected, which denotes that the significant *t*-values are placed outside the 95% confidence interval (CI). More than ten successively significant windows (duration of ≥60 ms (4 ms × 10 windows + 20 ms)) were reported as surface ERP effects.

### 2.6. CSD Analysis

We also estimated the cortical source activities or CSDs from the surface ERPs using sLORETA [43] and compared the CSDs between the SC and SI in each task. sLORETA ignores the high spatial resolution of neural electrical sources and instead solves the inverse problem of signal source estimation by finding the smoothest solution in the current direction and strength of the source grid points, i.e., it employs low spatial resolution to maximally avoid localization errors. This source estimation method is based on the basic concept of synchronization of adjacent neurons or neural assembly that occurs during sensory–perceptual and cognitive events. The spatial resolution of sLORETA covers the cortical gray matter and hippocampal areas, which are segmented into 6239 voxels with a size of 5 mm × 5 mm × 5 mm.

First, we obtained the three-dimensional (3D) coordinates of the 34 scalp electrodes. The 3D coordinates implemented by the EEG recording system were recalculated with reference to the no. 63 electrode placed at the vertex position. The 3D electrode coordinates were converted to Talairach coordinates, and a LORETA transformation matrix was created for the smoothest inverse solution in the current source estimation. Using a transformation matrix, each participant’s surface ERP data were transformed into CSD data (6239 voxels × 250 temporal points) for the SC and SI in each task. For signal source transformation, we used the sLORETA software (version 20081104; http://www.uzh.ch/keyinst/loretaOldy.htm (accessed on 22 February 2019)).

To statistically test semantic congruency effects in each task, we compared CSDs between the SC and SI at each voxel using a paired *t*-test. Due to the large number of comparisons (6239 voxels × 195 time windows (0–800 ms post-stimulus) = 1,216,605), we also performed a permutation *t*-test in a moving-average manner with a 20-ms interval (4-point overlap). Current source density data (6239 voxels × 245 time windows × 12 persons × 2 conditions = 36,685,320) were first normalized into *z*-scores and randomly resampled to obtain 12 dummy samples for the SC and SI. Resampling was repeated 100,000 times (approximately 1,216,605 comparisons/10 successively significant intervals) to produce a permutation *t*-distribution. Actual *t*-values outside the 95% CI of the distribution were considered as significant at the α level of *p* < 0.05, corrected. Based on the criterion of ≥ 10 successive significant time windows being considered as an effective source activity, data from voxels not satisfying the criterion were discarded from subsequent analyses. The *t*-value matrices for each task group (surviving voxels × 195 windows) were converted into binary data (0 = no significance (*p* > 0.05, uncorrected); 1 = significance (*p* ≤ 0.05, uncorrected)) and were hierarchically clustered using Euclidean distance and Ward’s method to obtain voxel clusters with similar temporal activation patterns. Based on the 3D coordinate information of voxels within each cluster, Brodmann areas (BAs) comprising more than five adjacent voxels were listed as task-specific neural correlates, and the cortical source locations were mapped onto a 3D whole-brain model using the CONN toolbox (version CONN20.b; http://www.nitrc.org/projects/conn,(accessed on 22 February 2019). RRID:SCR_009550).

### 2.7. Dynamic FC and Neural Coupling Analyses

This analysis aimed to examine whether frontoparietal FC was differentially related to source activity in the right cortical areas in the EST and IST. Before the dynamic FC analysis, we specified the frontal and parietal areas commonly activated for semantic congruency processing in both the EST and IST as follows: for each temporal point, averaged CSDs and standard deviations for the SC were calculated across the 6239 voxels; voxels over the threshold > max CSD minus 1.5 *SD* were specified for the EST and IST. Voxels were discarded if they did not yield any above-threshold activity throughout the overall time course, and the surviving voxels overlapping in the EST and IST were identified.

Dynamic FCs were calculated between the overlapping BAs in the frontal and parietal regions for each participant using a moving time window or SWC. Twelve temporal points (48 ms) in each parietal BA were correlated with the corresponding temporal points in each frontal BA using a sliding window with a 11-point overlap (189 time windows (0–800 ms post-stimulus)) for each participant.

Subsequently, we examined the coupling between frontoparietal FCs and source activity in the right temporal area during congruent semantic processing (SC). For each participant, Pearson’s correlation analyses were conducted between frontoparietal FCs and source activity in the right temporal BAs (400–700 ms) for each 100 ms interval. Source activities in the right temporal BAs were obtained in the following manner: The current source activities of each voxel of each BA were first normalized across the SC and SI and averaged separately for each participant. The average CSD for the SI was subtracted from that for the SC as the semantic congruency effect. Differences in the right temporal CSDs during each 100-ms interval (25 temporal points) were correlated with the corresponding frontoparietal FCs for each participant.

Finally, we statistically tested the differences in the neural coupling strength between the EST and IST. To explore when and which frontoparietal FCs yielded significant differences in correlation strength with the right temporal CSDs between the EST and IST, mixed analyses of variance (ANOVAs) were conducted using the within-participant factor of right temporal BA (nine BAs) and the between-participant factor of task (EST and IST). An initial ANOVA was applied separately to each temporal interval (400–500 ms, 500–600 ms, and 600–700 ms) and frontoparietal FC (5 parietal BAs × 2 frontal BAs). When a significant interaction was observed, a post hoc ANOVA with the task factor was conducted for each right temporal BA. The correlation coefficients (*r*) indicating frontoparietal FCs were transformed into Fisher *z*-scores. The statistical significance was determined using permutation tests. Upon the initial ANOVA, data (9 right temporal BAs × 12 participants × 2 tasks = 216 samples) were first randomized for each temporal interval and frontoparietal FC, and 216 samples were recollected to produce a data matrix of 9 temporal BAs × 24 participants. The resampling procedure was repeated 10,000 times to produce dummy *F*-values for each ANOVA. The dummy *F*-values (10,000 re-samplings × 10 frontoparietal FCs × 3 temporal intervals) were merged to produce a summary *F*-distribution (*n* = 300,000). Actual *F*-values were considered significant at the α level *p* < 0.05, corrected, when placed outside the 95% CI of the permutation *F*-distribution. For post hoc ANOVAs for the main effect of the task in each right temporal BA, data (12 participants × 2 tasks = 24 samples) were first randomized for each significant temporal interval and each significant frontoparietal FC, and 24 samples were recollected to produce a data matrix (12 participants × 2 tasks). Dummy *F*-values were produced 10,000 times for each test. Actual *F*-values were considered significant at the α level of *p* < 0.05, corrected, when placed within the upper 5% of the permutation *F*-distribution.

## 3. Results

### 3.1. Results of Surface Potential Analysis

**Figure 2:**
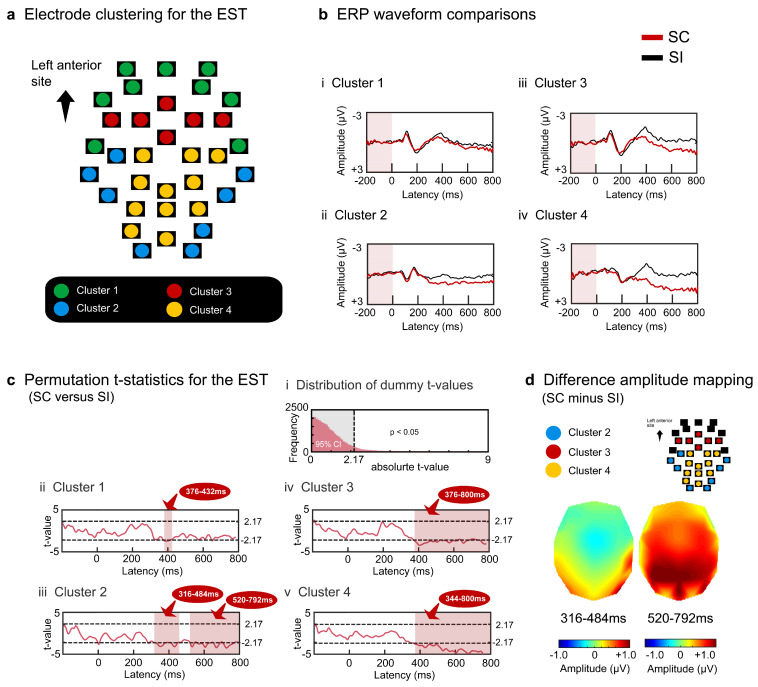
Surface potential analysis results for the EST. The grand-averaged ERP waveforms of the 34 electrodes were initially grouped into four clusters based on the hierarchical clustering method (**a**). The waveforms in each cluster were averaged for each participant, and the grand-averaged waveforms for the four clusters were compared between the SC and SI (**b**). Amplitude differences between the SC and SI were statistically tested using permutation *t*-tests (**c**). The light red-colored areas indicate intervals with significant differences between the two conditions (|*t*| > 2.17, *p* < 0.05 corrected). Scalp distributions of the difference amplitudes (SC–SI) were mapped for the two intervals (**d**). The dark red-colored areas represent more positive amplitudes.

The electrodes were separated into four superordinate clusters based on the semantic congruency effects in the EST (Figure 2a). Cluster 1 (green) was located in the most anterior frontal area with no significant differences between the waveforms for the SC and SI (Figure 2bi). Cluster 2 (blue) was observed at the edge of the bilateral posterior sites and showed reduced negative potentials for the SC (priming ERP effect) at approximately 300 ms post-stimulus (Figure 2bii). Cluster 3 (red) was located in the frontocentral area, with reduced negative potentials for the SC at approximately 400 ms post-stimulus (Figure 2biii). Cluster 4 (yellow) appeared in the midline central posterior areas, showing reduced negative potentials for the SC at approximately 300 ms post-stimulus (Figure 2biv).

Permutation *t*-tests specified the absolute *t*-value threshold of 2.17 (Figure 2ci). We subsequently performed paired *t*-tests between the SC and SI using a moving time window (20-ms interval with a 4-point overlap) and found that the above-threshold windows comprised ten or more successively significant effects. All electrode clusters showed above-threshold intervals of approximately 300 ms post stimulus. Cluster 1 transiently showed significant effects around 400 ms (376–432 ms, 2.20 < *ts* < 2.43, *ps* < 0.05 corrected) (Figure 2cii). Cluster 2 yielded significant effects after around 300 ms (316–484 ms, 520–792 ms, 2.18 < *ts* < 3.50, *ps* < 0.05 corrected) (Figure 2ciii). Clusters 3 and 4 similarly sustained significant effects after about 400 ms (cluster 3: 376–800 ms, 2.17 < *ts* < 3.45, *ps* < 0.05 corrected; cluster 4: 344–800 ms, 2.24 < *ts* < 5.29, *ps* < 0.05 corrected) (Figure 2civ,cv). In summary, the reduced negative SC effects in the EST were sustained after approximately 400 ms at the bilateral posterior sites (Figure 2d).

**Figure 3:**
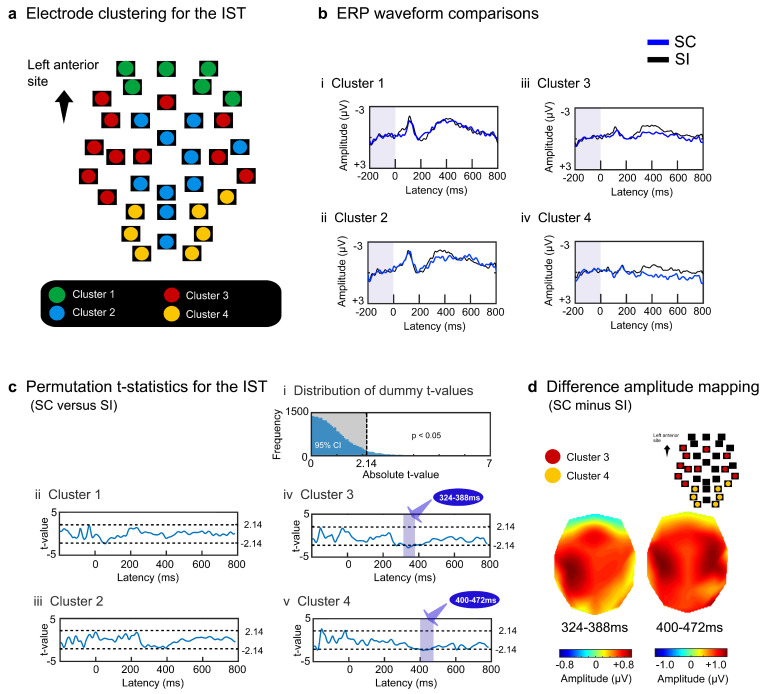
Surface potential analysis results for the IST. The grand-averaged ERP waveforms of the 34 electrodes were grouped into four clusters similar to the EST (**a**). The waveforms in each cluster were averaged for each participant, and the grand-averaged waveforms for the four clusters were compared between the SC and SI (**b**). Amplitude differences between the two conditions were tested using permutation *t*-tests (**c**). The light blue-colored areas indicate intervals with significant differences between the two conditions (|*t*| > 2.14, *p* < 0.05 corrected). Scalp distributions of the difference amplitudes were mapped for the two intervals (**d**). The dark red-colored areas represent more positive amplitudes.

For the IST, the four electrode clusters were separated with regard to semantic congruity ERP effects (Figure 3a). Cluster 1 (green) was located at the most anterior frontal site and did not show a significant difference between the SC and SI (Figure 3bi). Cluster 2 (blue) appeared at the midline centroposterior site, yielding reduced negative potentials for the SC at approximately 400 ms (Figure 3bii). Cluster 3 (red) was located at the bilateral temporal sites, yielding transient reduced negative potentials for the SC at approximately 400 ms (Figure 3biii). Cluster 4 (yellow) appeared at the most posterior sites, including the occipital areas, and showed reduced negative potentials for the SC after approximately 400 ms (Figure 3biv).

Permutation *t*-tests calculated the absolute *t*-value at the threshold of *p* < 0.05 and obtained a border value of 2.14 (Figure 3ci). For each electrode cluster, we performed paired *t*-tests with the observed data to compare the SC and SI using moving time windows. Clusters 1 and 2 did not yield any intervals with a significant difference between the SC and SI (Figure 3cii,ciii). Clusters 3 and 4 yielded significant intervals from about 300 to 500 ms (cluster 3: 324–388 ms, 2.15 < *ts* < 2.82, *ps* < 0.05 corrected; cluster 4: 400–472 ms, 2.14 < *ts* < 2.64, *ps* < 0.05 corrected) (Figure 3civ,cv). Cluster 3 generated reduced negative potentials predominantly at the left central lateralized sites during earlier temporal intervals (the potential map on the left in Figure 3d). These effects extended to the sites in the contralateral hemisphere at later intervals (the potential map on the right in Figure 3d).

To summarize, explicit semantic processing continued to show reduced negative effects in the midline and posterior areas, whereas implicit semantic processing transiently produced reduced potential effects in the lateral sites.

### 3.2. Results of the Signal Source Analysis

**Figure 4:**
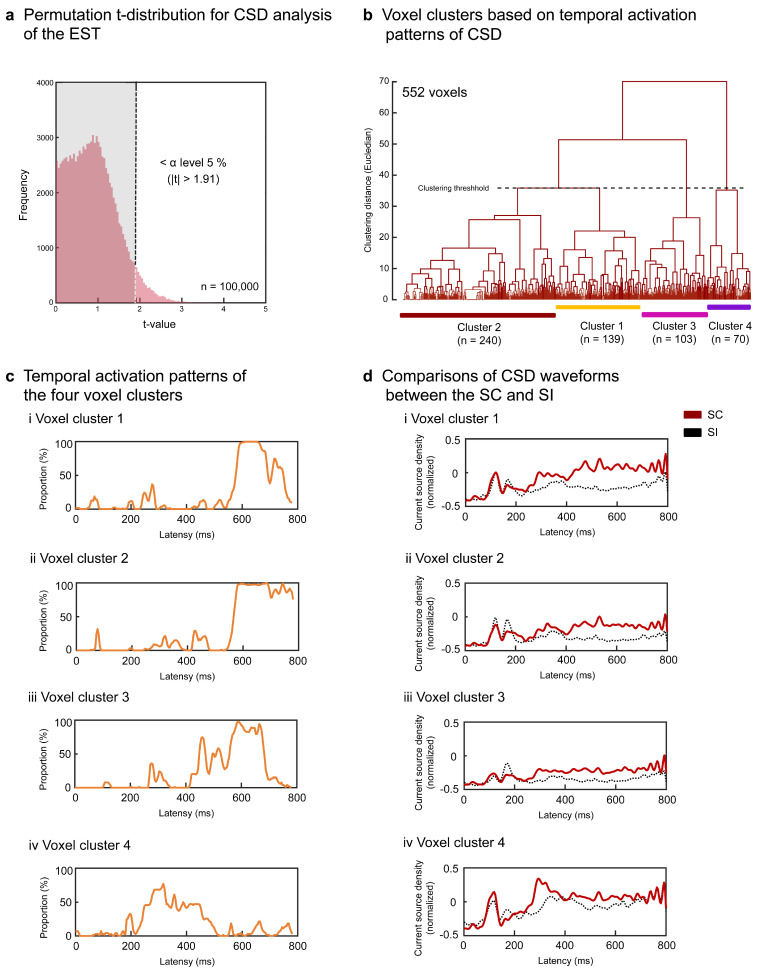
Signal source analysis results for the EST. Current source density waveforms of each voxel were statistically compared between the SC and SI for each moving temporal interval (20 ms with a 4-point overlap). Significant differences were determined based on the permutation distribution of dummy *t*-values (**a**). Voxels without ≥10 successively significant intervals were discarded from the subsequent analyses. The *t*-values of the remaining 552 voxels were converted into binary data (0 or 1) and separated into four clusters (**b**). The population ratios of voxels with the value 1 are represented in the temporal sequence for each cluster, denoting the temporal activation pattern of each cluster (**c**). The CSD waveforms of each cluster were compared between the SC and SI (**d**).

**Figure 5:**
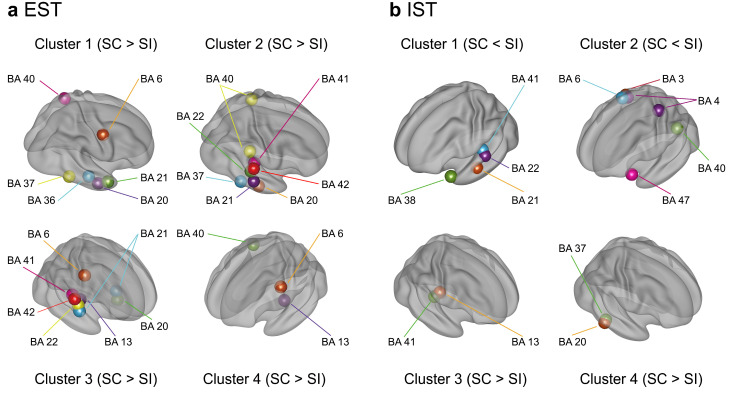
Three-dimensional cortical mapping of BAs that exhibited significant differences in activation between the SC and SI in the EST (**a**) and IST (**b**). Three-dimensional coordinates of each BA are determined by averaging the coordinates of voxels with the same BA label in each cluster. The numbers of the four clusters correspond to those previously shown in Figure 4 and Figure 6 for the EST and IST, respectively. For the EST, the right temporal BAs showed greater activation for the SC than for the SI (clusters 1 to 4). For the IST, the left superior and middle temporal areas (BAs 21, 22, 38, and 41) exhibited reduced activation for the SC than for the SI in earlier intervals (cluster 1). Conversely, several right superior and inferior temporal areas (BAs 20 and 41) showed greater activation for the SC than for the SI during later intervals (clusters 3 and 4).

For EST, each participant’s surface potentials of the SC and SI were transformed into CSD data (6239 voxels × post-stimulus 200 points (0–800 ms)). To specify the significant voxels for the SC effect, we conducted permutation *t*-tests using moving temporal windows (20 ms with a 4-point overlap) and determined the *t*-value threshold of >1.91 at the corrected α level of *p* < 0.05 (rank of *t*-value > 95,000) (Figure 4a). After discarding voxels based on the predefined criterion (<10 successively significant windows), 552 voxels (8.9%) were separated into four superordinate clusters (1.91 < *ts* < 5.29, *ps* < 0.05 corrected) (Table 1 and Figure 4b). The temporal activation patterns of the four clusters are represented by the population ratios (%) of significant voxels (number of significant voxels/total number of voxels × 100 for each cluster) in Figure 4c. All clusters showed greater activation for the SC than for the SI. Voxels in clusters 1 and 2 exhibited greater activation for the SC than for the SI after approximately 500 ms (Figure 4ci,cii,di,dii) and widely included the right temporal areas (clusters 1 and 2 in Table 1 and Figure 5a). Voxels in cluster 3 showed greater activation for the SC than for the SI during intervals of approximately 400–700 ms (Figure 4ciii,diii). This cluster included the left inferior/middle temporal areas (BAs 20 and 21), right superior/middle temporal areas (BAs 21, 22, 41, and 42), right precentral or premotor area (BA 6), and right insula (BA 13) (cluster 3 in Figure 5a). Cluster 4 transiently yielded greater activation for the SC than the SI from about 200 to 400 ms (Figure 4civ,div) in the left precentral gyrus (BA 6), left insula (BA13), and right inferior parietal area (BA 40) during earlier intervals (cluster 4 in Figure 5a).

**Figure 6:**
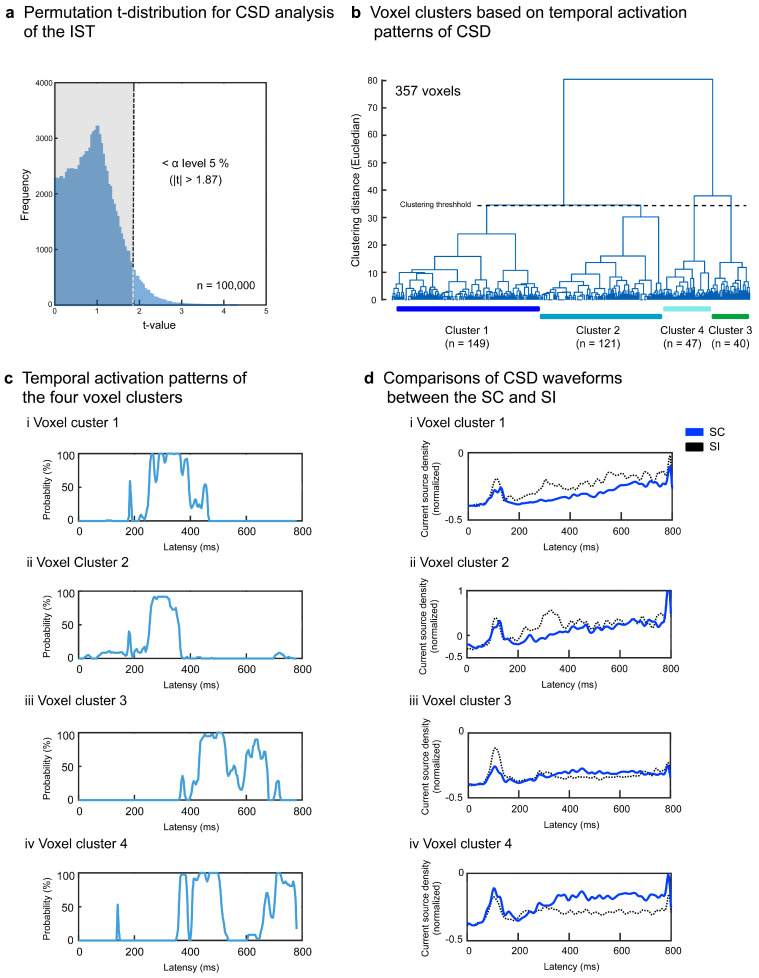
Signal source analysis results for the IST. Current source density waveforms of each of 6329 voxels were statistically compared between the SC and SI for each moving temporal interval (20 ms with a 4-point overlap). Significant differences were determined based on the permutation distribution of dummy *t*-values (**a**). Voxels without ≥10 successively significant intervals were discarded. The *t*-values of the remaining 357 voxels were separated into four clusters (**b**). The population ratios of voxels with significant differences between the SC and SI are represented in the temporal sequence for each cluster (**c**). The CSD waveforms of each cluster are compared between the SC and SI (**d**).

Regarding the IST, the permutation test determined the *t*-value threshold > 1.87 at the α level of *p* < 0.05, corrected (Figure 6a). After discarding voxels without 10 successively significant windows, 357 voxels (5.7%) survived and were separated into four superordinate clusters (1.87 < *ts* < 4.03, *ps* < 0.05 corrected) (Table 2 and Figure 6b). The temporal activation patterns of the four clusters are represented by the population ratios of the significant voxels in Figure 6c. Clusters 1 and 2 showed lower activation for the SC than for the SI around 300 ms (Figure 6ci,cii,di,dii) mainly in the left anterior superior/middle temporal areas (BAs 21 and 38), left superior/middle temporal areas (BAs 22 and 41), and left inferior frontal area (BA 47) (clusters 1 and 2 in Figure 5b). Clusters 3 and 4 demonstrated greater activation for the SC than for the SI after about 400 ms (Figure 6ciii,civ) in the right superior temporal area (BA 41), right inferior temporal areas (BAs 20 and 37), and right insula (BA 13) (clusters 3 and 4 in Figure 5b).

To summarize, explicit semantic processing resulted in a greater activation of the right temporal areas for the SC than for the SI during later intervals. Implicit semantic processing led to a lower activation of mainly the left anterior temporal areas during earlier intervals for the SC than for the SI but showed a greater activation of several right temporal areas during later intervals for the SC than for the SI.

### 3.3. Results of Dynamic FC and Neural Coupling Analyses

**Figure 7:**
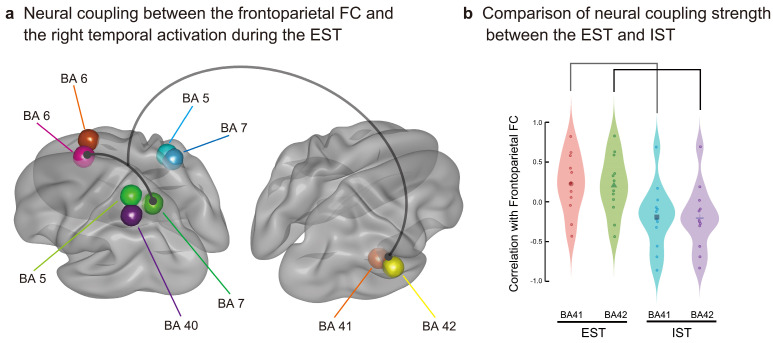
Increased neural coupling between the left frontoparietal FC (BAs 6 and 7) and right superior temporal activity (BAs 41 and 42) during the EST (**a**). Correlation strength is significantly higher for the EST than for the IST during the later interval, from 600 to 700 ms (**b**).

We first specified the globally activated voxels among the 6239 voxels using grand-averaged CSD data of the SC for the EST and IST. Based on the threshold > max CSD minus 1.5 *SD*, 609 (9.6%) and 290 voxels (4.6%) were extracted for the EST and IST, respectively. As summarized in Table 3, we identified 152 overlapping voxels in the EST and IST in the bilateral frontal and parietal areas. Subsequently, we conducted a dynamic FC analysis for each participant, and correlation coefficients (*r*) were calculated between each of the five parietal areas (bilateral BAs 5 and 7 and left BA 40) and each of the two frontal areas (bilateral BAs 6) using the SWC method (189 moving intervals). Using the time series of the frontoparietal dynamic FCs, we further calculated the correlation coefficients or neural coupling strengths between the FCs and source activities in the nine right temporal BAs (cluster 2 activated after about 500 ms during the EST; Figure 5a and Table 4) for each participant. Using the calculated coefficients, we conducted mixed ANOVAs with the right temporal BA (nine areas) as the within-participant factor and the task (EST and IST) as the between-participant factor to statistically test for differences in the neural coupling strength between the tasks. Initial ANOVAs demonstrated a significant interaction (right temporal BA × task: *Fs*(8,176) > 1.991, *ps* < 0.05 corrected) for the seven frontoparietal FCs at the interval of 600–700 ms (Table 5). As shown in Figure 7a and Table 6, follow-up ANOVAs showed higher correlations (*Fs*(1,22) > 4.274, *ps* < 0.05 corrected) between the frontoparietal FC between the left BAs 6 and 7 and CSDs in the two right superior temporal areas (BAs 41 and 42) for the EST than for the IST (600–700 ms: task, BA 41, *F*(1,22) = 6.363, *p* = 0.018 corrected; BA 42, *F*(1,22) = 6.112, *p* = 0.020 corrected) (Figure 7b). These results indicate that explicit semantic processing increased the neural coupling between the frontoparietal FC and right superior temporal activity during the later processing interval.

## 4. Discussion

The current study examined how interhemispheric and frontoparietal FCs interact during explicit semantic processing using an EEG. We hypothesized that explicit semantic processing promotes neural activity in nondominant right hemispheric areas by synchronizing with frontoparietal FC. As predicted, explicit semantic processing enhanced the neural coupling between right temporal activity and frontoparietal FC during later processing stages.

### 4.1. Results of Surface Potential Analysis

Semantic congruency effects differed between the EST and IST. Congruent semantic processing in the EST continued to have positive posterior-dominant effects during later intervals. In contrast, congruent semantic processing in the IST yielded lateralized positive amplitude effects for shorter durations. The EST drives complex controlled processing, such as prediction of target words [51,52], semantic integration [53,54], and, as more recently argued, controlled semantic cognition [55,56]. In contrast, the IST promotes automatic semantic processing by spreading neural activation across feature representations [38,39,40]. Neural activation decays rapidly without attention. Therefore, surface ERP effects in the IST did not persist for longer durations.

### 4.2. Results of CSD Analysis

The signal source analysis findings provide spatiotemporal information regarding the differences in cortical activity between explicit and implicit semantic processing, which cannot be elucidated by surface ERP analysis. During the EST, the source activity for the SC increased in the nondominant right temporal areas during later temporal windows. During the IST, the source activity at approximately 300 ms decreased for the SC in the left anterior/superior temporal and inferior frontal areas; conversely, the latter source activity increased for the SC in limited right superior/inferior temporal areas.

The asymmetry in the activity in the left anterior temporal and inferior frontal areas between the EST and IST demonstrates the changes in automaticity of semantic processing. These two areas are anatomically connected via the uncinate fasciculus or extreme capsule [7,57,58]. The left anterior temporal area (BA 38) is related to semantic integration [6]. The features activated as a concept unit are computed for matching or coherence [59] via an amodal neural hub [56,60]. The inferior frontal area (BA 47), on the other hand, is associated with the activation or selection of appropriate features [61,62,63,64]. The present study demonstrates that the left frontotemporal connection can be automatically activated for semantic processing without direct attention to semantic relations. This is because the SC included semantically congruent primes and targets, and the activated features might be matched promptly and integrated easily for semantically congruent pairs in the IST. In the EST, on the other hand, its features, irrespective of semantic congruency, might be activated similarly for the SC and SI, and feature activation may be maintained under attention. In summary, frontotemporal connections modulate the neural activity for semantic processing, hinging on attentional allocation.

The right temporal areas were widely activated for congruent semantic processing during later intervals of the EST. These temporal areas included the primary auditory cortex (BAs 41 and 42), left superior/middle temporal areas (BAs 21 and 22), and left inferior temporal areas (BAs 20 and 37). Considering the functions of the homologous areas in the left hemisphere, attentional semantic processing recruits a variety of verbal processing, which includes auditory sensory processing in the primary auditory cortex (BAs 41 and 42) [65,66], sound–lexical mapping (middle temporal gyrus: BA 21) [67,68], morpho-syntactic processing [68,69] of prime nouns and target verbs (anterior/superior temporal gyrus: BA 22), and lexical–semantic processing (inferior temporal gyrus: BAs 20 and 37) [70]. Consistent with our results, a meta-analysis [10] reported that phonological, lexicosemantic, and sentential processing activate the right superior temporal areas (BAs 41 and 42), posterior portions of the superior, middle, and inferior temporal areas (BAs 21 and 22), and anterior and posterior superior/middle temporal (BAs 22 and 21) areas, respectively. The left and right temporal areas are connected via the posterior part of the corpus callosum (splenium) and anterior commissure [4,7,71]. Direct attention to semantic associations influences the various stages of verbal processing, supported widely by nondominant temporal areas.

### 4.3. Results of Neural Coupling Analysis

Semantic congruency processing during the EST and IST bilaterally activated the superior frontal (BA 6) and superior parietal (BAs 5 and 7) areas in an overlapping manner. However, this bilateral frontoparietal FC was more activated in the EST than in the IST and may be relevant to a dorsal attentional network [72,73]. This dorsal network bilaterally connects the dorsal prefrontal (e.g., BA 8) and superior parietal (e.g., BA 7) areas for top-down visual attention [73]. The EST likely encouraged participants to predict and visually attend to semantically associated targets, strengthening the dorsal frontoparietal FC.

Left frontoparietal FC showed a significantly higher positive correlation with source activity in the right temporal areas for the EST than for the IST; these right temporal areas were restricted to the primary auditory cortex (BAs 41 and 42). Left frontoparietal FC, increased by visual word attention, was assumed to promote nondominant auditory processing in a cross-modal manner. This suggests that phonological processing, such as selective phonological attention [74,75] and phonological repetition [76], was reproduced for recalling congruent primes to help high-load semantic judgement during later processing stages, with neural coupling between the frontoparietal FC and neural activation in the right primary auditory areas.

### 4.4. Limitations

There are several methodological limitations in the present study. First, the spatial resolution of our electrode array is too sparse to estimate more precise signal source locations. Although LORETA conducts spatial smoothing to localize intracranial signal sources for surface potentials, more high-resolved spatial sampling (e.g., 256 scalp electrodes) avoids spatial aliasing and helps us to detect more dense source activities and functional connectivity [77]. Second, LORETA is a classical algorithm, compared to a novel Bayesian algorithm, such as thin Dugh [78]. LORETA, compared to novel algorithms, may estimate more dispersed neural sources and be vulnerable in detecting source activities with lower frequency and signal-to-noise levels [78]. More novel algorithms may enable us to detect more localized source activity for semantic processing, which, however, is beyond the capacity of the author. Third, the present method (SWC) for calculating dynamic FC is not unique and the most superior among various methods, as suggested by one of the reviewers. Other techniques, such as power envelope correlation, should be examined to extract rigorous FCs, if under a single task condition. It should be noticed, however, that the dominant aim of the present study is to compare neural coupling strengths between the EST and IST: The frontoparietal FC was calculated for the EST and IST based on the same method, and the neural coupling strength was higher in the EST than in the IST. The difference in relative strength between the two tasks may be more fundamental for the aim of the present study.

## 5. Conclusions

The present findings demonstrate that enhanced spatiotemporal neural coupling between general-cognitive and language-associated neural resources is related to explicit semantic processing. Furthermore, the neural coupling was cross-modal, in that the visual-based frontoparietal network was associated with enhanced neural activation of phonological representations based in the nondominant primary auditory cortex.

Cross-sectional, interhemispheric connectivity is mainly related to a domain-specific function, which can occur automatically in a dominant hemisphere. Remote longitudinal connectivity across different lobes contributes to bridging different functions. Higher-order information processing likely promotes the dynamic neural coupling between cross-sectional and longitudinal connectivity, frequently accompanied by conscious awareness. Future studies should investigate the neural correlates or origins that establish such crisscross neural coupling for mysterious, conscious information processing.

## Figures and Tables

**Table 1:** Voxel clusters showing activation for the EST.

Cluster	Cortical Area	BA	Numbers of Voxels	MNI Coordinates					
				x		y		z	
				*M*	*SD*	*M*	*SD*	*M*	*SD*
1	R. precentral/middle frontal gyrus	6	7	45.7	6.1	−5.0	4.1	44.3	3.5
	R. inferior temporal/fusiform gyrus	20	25	38.2	9.0	−11.6	15.9	−35.0	9.5
	R. middle temporal gyrus	21	37	51.1	7.6	−0.3	6.8	−26.6	8.7
	R. parahippocampal gyrus	36	8	32.5	3.8	−23.8	9.2	−27.5	7.6
	R. fusiform gyrus	37	14	45.4	7.7	−51.4	6.6	−17.9	6.1
	L. inferior parietal gyrus	40	21	−42.4	5.2	−47.9	4.4	51.7	4.0
2	R. inferior temporal/fusiform gyrus	20	82	50.0	7.8	−24.0	13.7	−27.2	7.0
	R. middle temporal gyrus	21	56	62.3	5.0	−29.7	9.6	−9.1	5.9
	R. middle/superior temporal gyrus	22	30	59.0	7.2	−34.0	7.1	3.5	5.3
	R. inferior temporal/fusiform gyrus	37	25	52.0	4.8	−47.4	5.2	−19.6	4.8
	L. inferior parietal gyrus	40	8	−41.9	5.9	−40.0	3.8	45.6	4.2
	R. inferior parietal/postcentral gyrus	40	8	48.1	10.3	−36.3	9.9	30.6	12.1
	R. superior temporal gyrus	41	11	48.6	5.0	−30.9	3.8	10.0	3.9
	R. superior temporal gyrus	42	10	62.5	4.9	−29.0	3.2	11.5	4.1
3	R. precentral gyrus	6	6	46.7	5.2	−8.3	2.6	39.2	3.8
	R. insula/superior temporal gyrus	13	9	43.3	2.5	−16.1	8.6	3.9	8.6
	L. inferior temporal gyrus	20	9	−59.4	3.9	−20.0	4.3	−25.6	5.3
	L. middle temporal gyrus	21	19	−59.2	6.5	−22.1	5.4	−13.2	4.5
	R. middle temporal gyrus	21	11	56.4	9.2	−9.5	6.1	−7.7	4.1
	R. superior temporal gyrus	22	13	56.5	8.5	−11.2	4.2	0.4	3.8
	R. superior temporal gyrus	41	11	46.8	5.6	−25.0	5.0	10.0	3.2
	R. superior temporal gyrus	42	7	61.4	3.8	−15.0	4.1	10.0	0.0
4	L. precentral gyrus	6	16	−50.0	7.5	−5.3	3.9	35.6	3.1
	L. insula	13	10	−41.0	3.2	−18.0	4.8	6.0	3.2
	R. inferior parietal/supramarginal gyrus	40	31	59.7	3.9	−44.0	4.7	34.0	8.2

MNI: Montreal Neurological Institute; BA: Brodmann area; L: left; R: right.

**Table 2:** Voxel clusters showing activation for the IST.

Cluster	Cortical Area	BA	Numbers of Voxels	MNI Coordinates					
				x		y		z	
				*M*	*SD*	*M*	*SD*	*M*	*SD*
1	L. middle temporal gyrus	21	50	−57.0	7.8	−15.6	16.7	−15.6	13.2
	L. middle/superior temporal gyrus	22	22	−59.1	5.0	−26.8	9.2	2.0	3.3
	L. superior temporal gyrus	38	52	−38.3	7.9	13.5	4.6	−28.3	7.5
	L. superior temporal gyrus	41	8	−53.1	2.6	−25.0	3.8	8.1	2.59
2	R. postcentral gyrus	3	12	36.7	4.9	−28.8	4.3	55.4	5.0
	L. precentral gyrus	4	18	−34.3	7.3	−27.1	7.6	62.1	7.6
	R. precentral gyrus	4	11	31.4	3.2	−26.8	4.0	56.8	6.8
	R. precentral/middle frontal gyrus	6	17	27.1	3.1	−14.4	5.0	60.9	7.3
	L. inferior parietal/supramarginal gyrus	40	24	−53.3	8.6	−43.1	3.6	37.9	8.2
	L. inferior frontal gyrus	47	13	−31.5	6.6	17.3	3.9	−19.6	3.2
3	R. insula	13	25	37.8	5.0	−23.4	5.9	15.4	4.3
	R. superior temporal gyrus	41	13	42.7	3.3	−29.6	5.6	9.2	3.4
4	R. inferior temporal gyrus	20	17	54.7	4.1	−44.7	6.7	−19.7	5.7
	R. inferior temporal/fusiform gyrus	37	22	50.7	5.8	−46.1	4.9	−18.6	4.9

MNI: Montreal Neurological Institute; BA: Brodmann area; L: left; R: right.

**Table 3:** Overlapping globally activated frontal and parietal areas in the EST and IST.

Cortical Area	Cortical Area Name	BA	Numbers of Voxels	MNI Coordinates					
				x		y		z	
				*M*	*SD*	*M*	*SD*	*M*	*SD*
Frontal	L. superior frontal gyrus	6	28	−10.5	5.8	8.9	10.7	66.6	3.6
	R. superior frontal gyrus	6	16	7.5	3.2	14.1	9.3	65.0	3.7
Parietal	L. postcentral gyrus	5	13	−29.2	6.4	−46.9	2.5	65.8	3.4
	R. postcentral gyrus	5	19	23.7	11.0	−47.1	2.5	66.3	3.7
	L. superior parietal gyrus/precuneus	7	46	−24.6	10.0	−61.7	6.6	59.7	7.6
	R. superior parietal/postcentral gyrus	7	16	21.3	10.1	−55.0	3.2	65.3	4.6
	L. inferior parietal gyrus	40	14	−40.7	4.3	−51.1	4.0	56.1	2.9

MNI: Montreal Neurological Institute; BA: Brodmann area; L: left; R: right.

**Table 4:** Right temporal areas used for the analysis of neural coupling with frontoparietal FC.

Right Temporal Area	BA	Numbers of Voxels	MNI Coordinates					
			x		y		z	
			*M*	*SD*	*M*	*SD*	*M*	*SD*
Fusiform gyrus	20	30	47.3	6.9	−25.7	11.1	−27.2	3.9
Inferior temporal gyrus	20	43	52.7	7.1	−23.1	15.1	−27.7	8.3
Middle temporal gyrus	21	52	62.9	4.5	−30.5	9.5	−9.1	5.8
Middle temporal gyrus	22	11	56.4	6.4	−36.4	3.9	1.4	2.3
Superior temporal gyrus	22	19	60.5	7.4	−32.6	8.2	4.7	6.1
Fusiform gyrus	37	14	50.0	3.9	−48.6	4.6	−20.7	4.7
Inferior temporal gyrus	37	11	54.5	4.7	−45.9	5.8	−18.2	4.6
Superior temporal gyrus	41	9	48.3	5.0	−31.7	3.5	10.0	4.3
Superior temporal gyrus	42	10	62.5	4.9	−29.0	3.2	11.5	4.1

MNI: Montreal Neurological Institute; BA: Brodmann area.

**Table 5:** Results of ANOVAs comparing the neural coupling strength between the EST and IST.

Parietal Area	Time Window (ms)	Task				Right Temporal Area × Task			
		L. BA 6		R. BA 6		L. BA 6		R. BA 6	
		*F*-Value	*p*-Value	*F*-Value	*p*-Value	*F*-Value	*p*-Value	*F*-Value	*p*-Value
L. BA 5	400–500	0.014	0.907	1.333	0.261	0.744	0.653	0.498	0.857
	500–600	1.658	0.212	0.660	0.425	0.075	1.000	0.319	0.958
	600–700	0.122	0.730	0.244	0.625	2.966 **	0.004	4.913 ***	<0.001
R. BA 5	400–500	0.103	0.751	0.038	0.847	0.471	0.876	0.741	0.656
	500–600	1.108	0.304	0.474	0.498	0.069	1.000	0.105	0.999
	600–700	0.556	0.463	0.005	0.944	1.572	0.136	1.409	0.196
L. BA 7	400–500	0.000	1.000	0.292	0.594	0.561	0.810	0.665	0.723
	500–600	0.735	0.401	2.425	0.134	1.083	0.377	0.424	0.906
	600–700	1.263	0.273	0.682	0.418	4.775 ***	<0.001	4.586 ***	<0.001
R. BA 7	400–500	0.167	0.687	0.903	0.352	0.459	0.884	0.719	0.676
	500–600	1.494	0.235	0.622	0.438	0.149	0.996	0.097	0.999
	600–700	0.019	0.891	0.252	0.620	1.300	0.247	3.529 **	0.001
L. BA 40	400–500	0.787	0.385	0.000	1.000	0.619	0.762	0.442	0.894
	500–600	0.222	0.642	0.069	0.795	0.810	0.596	0.685	0.705
	600–700	0.393	0.536	1.194	0.286	4.061 ***	<0.001	3.588 **	0.001

BA: Brodmann area; L: left; R: right; ** *p* < 0.01; *** *p* < 0.001.

**Table 6:** Results of follow-up ANOVAs of the neural coupling strength for each right temporal area between the EST and IST.

Right Temporal Area	Task					
	L. BA 6–L. BA 5		R. BA 6–L. BA 5		L. BA 6–L. BA 5	
	*F*-Value	*p*-Value	*F*-Value	*p*-Value	*F*-Value	*p*-Value
Fusiform gyrus (BA 20)	0.005	0.945	0.054	0.819	0.600	0.448
Inferior temporal gyrus (BA 20)	0.025	0.876	0.11	0.744	0.888	0.358
Middle temporal gyrus (BA 21)	0.502	0.487	0.667	0.424	2.376	0.139
Middle temporal gyrus (BA 22)	0.115	0.738	0.024	0.879	0.076	0.786
Superior temporal gyrus (BA 22)	0.206	0.655	0.208	0.654	0.014	0.907
Fusiform gyrus (BA 37)	0.041	0.842	0.07	0.794	0.032	0.860
Inferior temporal gyrus (BA 37)	0.513	0.482	0.59	0.451	2.489	0.130
Superior temporal gyrus (BA 41)	2.063	0.167	3.368	0.080	6.363	0.018 *
Superior temporal gyrus (BA 42)	1.594	0.222	2.524	0.127	6.112	0.020 *
**Right temporal area**	**Task**					
	**R. BA 6–R. BA 7**		**L. BA 6–L. BA 40**		**R. BA 6–L. BA 40**	
	***F*-value**	***p*-value**	***F*-value**	***p*-value**	***F*-value**	***p*-value**
Fusiform gyrus (BA 20)	0.514	0.482	0.133	0.720	0.841	0.370
Inferior temporal gyrus (BA 20)	0.476	0.498	0.229	0.638	1.088	0.310
Middle temporal gyrus (BA 21)	0.007	0.934	0.918	0.350	2.037	0.169
Middle temporal gyrus (BA 22)	1.502	0.235	0.008	0.930	0.236	0.633
Superior temporal gyrus (BA 22)	1.132	0.301	0.185	0.672	0.003	0.957
Fusiform gyrus (BA 37)	1.509	0.234	0.051	0.824	0.066	0.800
Inferior temporal gyrus (BA 37)	0.021	0.886	1.118	0.304	2.160	0.157
Superior temporal gyrus (BA 41)	0.510	0.484	2.414	0.135	3.724	0.066
Superior temporal gyrus (BA 42)	0.322	0.577	2.419	0.135	3.490	0.075

BA: Brodmann area; L: left; R: right; * *p* < 0.05.

## Data Availability

The data presented in this study are available on request from the corresponding author.
